# Anti-Neuroinflammatory *ent*-Kaurane Diterpenoids from *Pteris multifida* Roots

**DOI:** 10.3390/molecules22010027

**Published:** 2016-12-26

**Authors:** Jung Wha Kim, Ji Yeon Seo, Won Keun Oh, Sang Hyun Sung

**Affiliations:** College of Pharmacy and Research Institute of Pharmaceutical Science, Seoul National University, Seoul 08826, Korea; jkim11@snu.ac.kr (J.W.K.); quftkfka@gmail.com (J.Y.S.); wkoh1@snu.ac.kr (W.K.O.)

**Keywords:** *Pteris multifida*, *ent*-kaurane diterpenoids, anti-neuroinflammation, nitric oxide (NO), BV-2 microglia

## Abstract

Activated microglia are known to be a major source of cellular neuroinflammation which causes various neurodegenerative diseases, including Alzheimer’s disease. In our continuing efforts to search for new bioactive phytochemicals against neuroinflammatory diseases, the 80% methanolic extract of *Pteris multifida* (Pteridaceae) roots was found to exhibit significant NO inhibitory activity in lipopolysaccharide (LPS)-activated BV-2 microglia cells. Three new *ent*-kaurane diterpenoids, pterokaurane M_1_ 2-*O*-β-d-glucopyranoside (**4**), 2β,16α-dihydroxy-*ent*-kaurane 2,16-di-*O*-β-d-glucopyranoside (**10**), and 2β,16α,17-trihydroxy-*ent*-kaurane 2-*O*-β-d-glucopyranoside (**12**), were isolated along with nine other known compounds from *P. multifida* roots. The chemical structures of the new compounds were determined by 1D- and 2D-NMR, HR-ESI-MS, and CD spectroscopic data analysis. Among the isolates, compounds **1** and **7** significantly inhibited NO production in LPS-stimulated BV-2 cells reducing the expression of the cyclooxygenase-2 (COX-2) protein and the level of pro-inflammatory mediators such as prostaglandin E_2_ (PGE_2_), tumor necrosis factor (TNF)-α, interleukin (IL)-1β, and IL-6. These results suggest that *ent*-kaurane diterpenes from *P. multifida* could be potential lead compounds that act as anti-neuroinflammatory agents.

## 1. Introduction

Microglia are resident immune cells in the central nervous system [1]. In response to external stimuli such as lipopolysaccharides (LPS), they release various types of inflammatory molecules including nitric oxide (NO) [2]. In the brain, NO acts as a neuromodulator at synaptic junctions; however, high levels of NO produced by activated microglia induce oxidative stress and inflammation [3]. Neuroinflammation is associated with the pathogenesis and progression of neurodegenerative diseases such as Alzheimer’s disease, Parkinson’s disease and multiple sclerosis [4].

**Figure 1:**
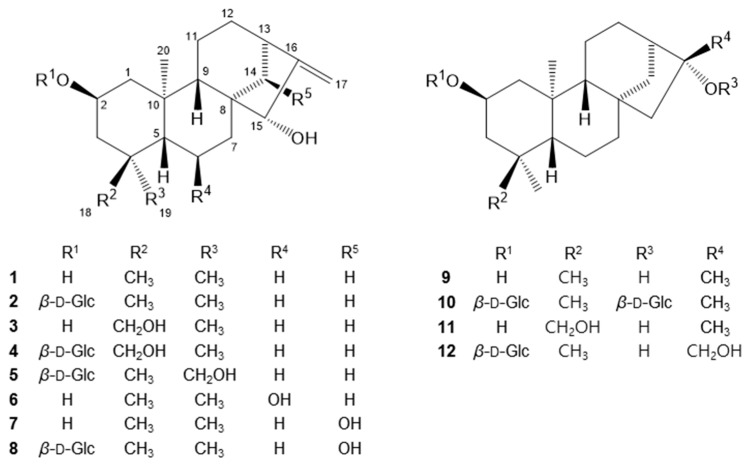
Chemical structures of the isolated compounds **1**–**12** from *P. multifida*.

During our continuing search for bioactive substances from medicinal plants that act against neuroinflammation, an 80% methanol extract of *Pteris multifida* Poir. (Pteridaceae) roots was found to significantly inhibit lipopolysaccharide (LPS)-induced NO production (IC_50_ 18.6 μg/mL). *P. multifida* is a perennial fern found in the southeast of China, Japan and South Korea [5,6]. As a folk medicine in China, the whole plant has been used to treat dysentery, cholecystitis, hepatitis, eczema, rheumatism, hematemesis, enteritis, and diarrhea [5,7]. Previous studies on the chemical constituents of *P. multifida* have revealed the presence of diterpenoids, sesquiterpenoids, flavonoids, coumarins, lignans, and sterols [5,6,7,8,9,10]. Recent studies have reported that the extract and constituents of *P. multifida* showed anti-tumor, anti-hyperlipidemic, and free radical-scavenging activities [11,12,13]. To the best of our knowledge, however, there have been no reports on any anti-neuroinflammatory activity of *P. multifida*. In the present study, we identified bioactive chemical constituents of *P. multifida* with NO inhibitory effects. We isolated 12 *ent*-kaurane diterpenoids **1**–**12** (Figure 1) including three new compounds, pterokaurane M_1_ 2-*O*-β-d-glucopyranoside (**4**), 2β,16α-dihydroxy-*ent*-kaurane 2,16-di-*O*-β-d-glucopyranoside (**10**), and 2β,16α,17-trihydroxy-*ent*-kaurane 2-*O*-β-d-glucopyranoside (**12**), and evaluated the NO inhibitory activity of all the isolates on LPS-activated BV-2 microglia cells.

## 2. Results and Discussion

### 2.1. Isolation and Characterization of the Ent-Kaurane Diterpenoids

**Figure 2:**
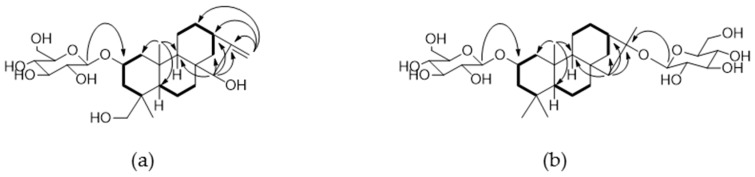
Key COSY (bold line) and HMBC (plain arrow) correlations of compounds **4** (**a**) and **10** (**b**).

**Figure 3:**
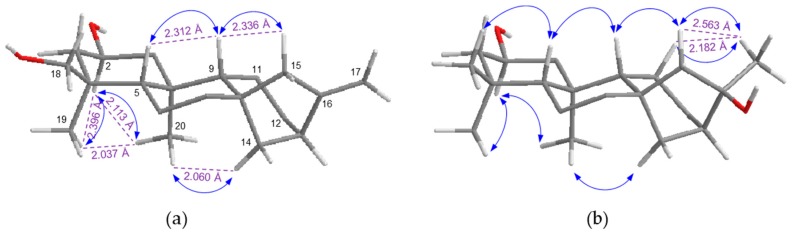
The NOESY correlations of each aglycone of compounds **4** (**a**) and **10** (**b**).

Compound **4** was isolated as a white amorphous powder, and the molecular formula, C_26_H_42_O_8_, was established by a HR-ESI-MS peak in the positive ion mode at *m*/*z* 483.2963 [M + H]^+^ (calcd. for C_26_H_43_O_8_, 483.2958) and by the NMR spectrum. Acid hydrolysis of **4** yielded glucose which had the same retention time as d-glucose in a HPLC experiment [14]. The ^1^H- and ^13^C-NMR spectrum of **4** showed resonances attributed to an olefinic group [δ_H_ 5.17 (1H, s), 5.06 (1H, s); δ_C_ 109.8], two tertiary methyl groups [δ_H_ 1.13 (3H, s), 0.79 (3H, s); δ_C_ 20.6, 19.5] and a β-glucopyranose group [δ_H_ 4.38 (1H, d, *J* = 7.8 Hz, H-1′); 103.3 (C-1′), 78.9 (C-5′), 78.6 (C-3′), 75.9 (C-2′), 72.5 (C-4′), 63.6 (C-6′)]. The ^13^C-NMR spectrum of **4** exhibited 20 carbon signals for an aglycone, which were resolved into two methyls (δ_C_ 20.6, 19.5), nine methylenes (δ_C_ 109.8, 72.7, 49.1, 43.0, 38.2, 36.8, 34.7, 20.7, 20.1), five methines (δ_C_ 84.6, 74.9, 56.6, 50.5, 44.5), and four quaternary carbons, suggesting that **4** contains an *ent*-kaurane diterpene skeleton. Comparisons of the ^13^C-NMR spectroscopic data of **4** with those reported for the *ent*-kaurane diterpene pterokaurane M_1_ (**3**) revealed extensive similarities, except for the additional signals of a glucose moiety [8]. The ^1^H–^1^H COSY and HMQC spectra of **4** showed the correlation signals of partial structural fragments of –CH_2_CHCH_2_– (C-1–C-2–C-3), –CHCH_2_CH_2_– (C-5–C-6–C-7), and –CH_2_CH_2_CHCH_2_– (C-11–C-12–C-13–C-14) (Figure 2a). The HMBC correlations from H-5 (δ_H_ 1.20, m) to C-1, C-4, C-9, C-10, CH_3_-19, and CH_3_-20 and from H-15 (δ_H_ 3.76, br s) to C-8, C-9, C-13, C-14, C-16, and C-17 support that **4** possesses an *ent*-kaurane skeleton (Figure 2a). Additionally, the correlations from H-17 [δ_H_ 5.07 (1H, s), 5.17 (1H, s)] to C-12, C-13, C-15, and C-16 reveal the presence of an exomethylene group on C-16, and the correlations from H-1′ (δ_H_ 4.38) to C-2 imply that the sugar moiety is attached to C-2. In the NOESY spectrum, the correlation signals from H-2 with CH_3_-19, 20 and from CH_3_-20 to H-14 reveal that the *O*-glucose group at C-2 is β-oriented, and the signals from H-17 to H-15a, H-15a to H-9, and H-9 to H-5 establish that the hydroxyl group at C-15 is α-oriented, respectively (Figure 3a) [15]. Moreover, the interatomic distances measured on the 3D structure of the aglycone of **4** in the ChemBio3D software support the above determination of the relative configurations (Figure 3a). The absolute configuration of **4** was elucidated from the CD spectra, which showed a negative cotton effect at 298 nm and a positive one at 325 nm, confirming that the stereochemistry of C-5 was a *S*-configuration (Figure S7) [15]. Based on the above data, compound **4** was determined to be pterokaurane M_1_ 2-*O*-β-d-glucopyranoside.

Compound **10** was obtained as a white amorphous powder, and its molecular formula was assigned as C_32_H_54_O_12_ according to the HR-ESI-MS peak at *m*/*z* 653.3516 [M + Na]^+^ (calcd. for C_32_H_54_O_12_Na, 653. 3513). The ^1^H- and ^13^C-NMR spectrum data of **10** showed the presence of an *ent*-kaurane diterpene skeleton exhibiting characteristic signals including four tertiary methyl carbons (δ_C_ 35.1, 23.5, 22.2, 20.2) and two β-d-glucopyranose moieties [δ_H_ 4.36 (2H, d, *J* = 7.8 Hz, H-1′, H-1″); δ_C_ 103.4, 100.1, 79.1, 78.9, 78.6. 78.4, 76.0, 75.9, 72.6, 72.5, 63.6, 63.5]. Comparison of the ^13^C-NMR data for the aglycone of **10** with that of **4** revealed quite similar patterns, except for the fact that **10** had a methyl signal at C-18 (δ_C_ 35.1) instead of an oxymethylene and exhibited no olefinic group at C-16 and C-17 but instead an oxygenated quaternary carbon (δ_C_ 89.3, C-16) with a tertiary methyl carbon (δ_C_ 22.2, C-17). When comparing the 1D and 2D-NMR data of **10** with its aglycone, the known compound 2β,16α-dihydroxy-*ent*-kaurane (**9**) [16], the two glucose groups are suggested to be linked to C-2 and C-16 from the significant downfield shift of C-2 (δ_C_ 75.0) and C-16 (δ_C_ 89.3) (Table 1 and Tables S1 and S2). The HMBC cross-peak correlations from the anomeric proton (δ_H_ 4.36 (2H, d, *J* = 7.8 Hz, H-1′, H-1″)) to C-2 and C-16 support the assignment above. The β-orientation of the *O*-Glc group at C-2 and the α-orientation of another *O*-Glc group at C-16 were determined by the NOESY correlations from H-2 to H-1a, H-3a, CH_3_-19, and CH_3_-20 and from CH_3_-17 to H-11b and H-15a, H-15a to H-9, H-9 to H-5, and H-5 to CH_3_-18, respectively (Figure 3b). Additionally, the calculated interatomic distances from CH_3_-17 to H-11b and H-15a on the 3D structure of the aglycone of **10** are 2.182 Å and 2.563 Å, respectively (Figure 3a), whereas the distances would be over the detection limit of the NOESY correlation (5 Å) when assuming the *O*-glucose group at C-16 has a β-orientation [17]. Thus, from the observation of the NOESY correlation, the location of where the glucose is attached and its relative configuration for this molecule were confirmed. The CD spectral data of **10** have features similar to those of **4**, which indicate that **10** has the same stereochemistry as **4** (Figure S14). Consequently, the structure of **10** was determined to be 2β,16α-dihydroxy-*ent*-kaurane 2,16-di-*O*-β-d-glucopyranoside.

Compound **12** was isolated as a white amorphous powder. The molecular formula of **12** was determined to be C_26_H_44_O_8_ based on the HR-ESI-MS signal at *m*/*z* 483.2931 [M − H]^−^ (calcd. for C_26_H_43_O_8_, 483.2958). Analysis of the ^1^H- and ^13^C-NMR data of **12** revealed similar patterns to those of compound **10** except for the presence of one glucose moiety (δ_C_ 103.4, 78.9, 78.6, 76.0, 72.5, 63.6) and an oxygenated methylene (δ_H_ 3.69 (1H, d, *J* = 11.4), 3.58 (1H, d, *J* = 11.4); δ_C_ 67.7). The HMBC correlations indicate that an oxymethylene is attached to the C-16 quaternary carbon (δ_C_ 83.6), and a glucose group is connected to C-2. The NOESY correlations from H-2 to H-1a, H-3a, CH_3_-19, and CH_3_-20 imply a β configuration for the 2-*O*-glucose, and the correlations from H_2_-17 to H-15a, H-15a to H-9, H-9 to H-5, and H-5 to CH_3_-18 establish an α configuration for the 16-OH. In addition, compound **12** had a similar CD spectra with that of **10** implying that they have the same absolute configurations (Figure S21). Based on the above data, **12** was determined to be 2β,16α,17-trihydroxy-*ent*-kaurane 2-*O*-β-d-glucopyranoside.

The nine known compounds were identified as 2β,15α-dihydroxy-*ent*-kaur-16-ene (**1**) [16], creticoside A (**2**) [16], pterokaurane M_1_ (**3**) [8], 2β,15α,19-trihydroxy-*ent*-kaur-16-ene 2-*O*-β-d-gluco-pyranoside (**5**) [18], 2β,6β,15α-trihydroxy-*ent*-kaur-16-ene (**6**) [18], pterokaurane P_1_ (**7**) [19], pterokaurane P_1_ 2-*O*-β-d-glucopyranoside (**8**) [19], 2β,16α-dihydroxy-*ent*-kaurane (**9**) [16], and pterokaurane R (**11**) [20] by comparing their spectroscopic data with values reported in the literature.

### 2.2. NO Inhibitory Effects of Ent-kaurane Diterpenoids

Some studies have reported on the anti-inflammatory activities of diterpenes from various plant extracts [21,22,23,24,25]. However, there have been no reports yet on the effects of *ent*-kaurane diterpenoids isolated from *P. multifida* on neuroinflammation. The characteristic feature of the *ent*-kaurane diterpenes isolated from *P. multifida* is the presence of a hydroxyl group at C-2 in their chemical structures. In this study, therefore, we evaluated the anti-neuroinflammatory activities of the isolated compounds **1**–**12** with LPS-activated BV-2 cells as the screening tool (Table 2).

Among the isolates, **1** and **7** showed significant inhibition of NO production in the LPS-activated BV-2 cells with an IC_50_ value of 13.9 μM and 10.8 μM, respectively, without any cytotoxicity. Especially, **1** and **7** showed higher NO inhibitory activities than that of previously reported *ent*-kaurane diterpenoids in BV-2 microglia cells [24]. It was shown that the compounds with a hydroxyl group at C-2 have more potent NO inhibitory activities than their glycosides (**1** > **2**, **3** > **4**, **7** > **8**, and **9** > **10**). In addition, the compounds with a hydroxyl group at C-6 exhibited decreased inhibitory activity against NO production (**1** > **6**). Therefore, the above data suggest that the NO inhibitory effect of the *ent*-kaurane diterpenoids is related to the degree of hydroxylation and substituted moieties.

**Figure 4:**
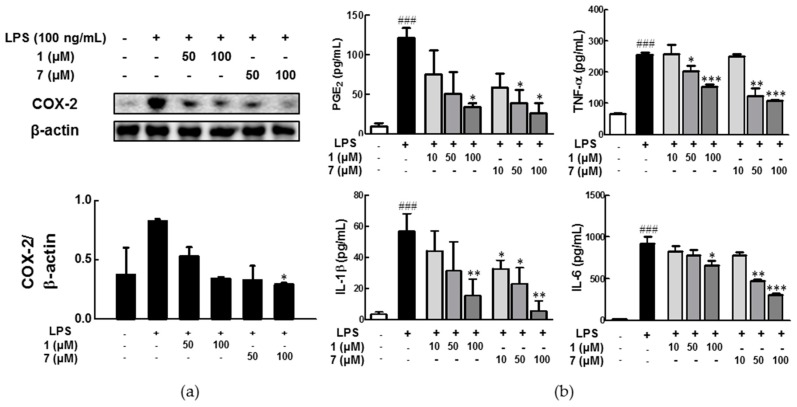
Anti-neuroinflammatory effects of compounds **1** and **7** in lipopolysaccharide (LPS)-activated BV-2 microglia. (**a**) The expression of cyclooxygenase (COX)-2 in LPS-activated BV-2 microglia in western blot. The data represent the mean ± SD. * *p* < 0.1 versus LPS-treated group (*n* = 2). Representatives of two independent experiments with similar results are shown; (**b**) The effects of compounds **1** and **7** on prostaglandin (PG)E_2_, tumor necrosis factor (TNF)-α, interlukin (IL)-1β, and IL-6 production in LPS-activated BV-2 microglia cells. ^###^ *p* < 0.001, compared to vehicle, * *p* < 0.05, ** *p* < 0.01, and *** *p* < 0.001, compared to the LPS-treated group (*n* = 3).

Because LPS is known to induce the production of pro-inflammatory mediators by cyclooxygenase-2 (COX-2) which is a pro-inflammatory enzyme that has a central role in the inflammatory response by converting arachidonic acids into prostaglandins (PGs) [26], the effects of **1** and **7**, which showed a significant NO inhibitory activity on LPS-induced COX-2 expression in activated BV-2 microglia cells, were evaluated by western blot analysis (Figure 4a). Our results show that the protein level of COX-2 increased by the LPS treatment was down-regulated by the treatments of **1** and **7**. In further examination of the effect of **1** and **7** on pro-inflammatory mediators, the level of cytokines including tumor necrosis factor (TNF)-α, interleukin (IL)-1β, and IL-6, and PGE_2_ enzyme in LPS-activated BV-2 microglia cells were effectively decreased by pre-treatment with **1** and **7** in a dose-dependent manner (Figure 4b). These results suggest that **1** and **7** suppress NO production by inhibiting COX-2 protein expression and pro-inflammatory mediators. To the best of our knowledge, this is the first report for the anti-neuroinflammatory effects of *ent*-kaurane diterpenoids from *P. multifida* in BV-2 microglia.

In summary, 12 *ent*-kaurane diterpene compounds **1**–**12**, including three new compounds **4**, **10**, and **12**, were isolated from the roots of *P. multifida*. Among the isolates, compounds **1** and **7** inhibit the production of NO and the pro-inflammatory mediators TNF-α, IL-1β, IL-6, and PGE_2_ as well as the expression of COX-2 in LPS-stimulated BV-2 microglia cells. Although an in-depth in vivo study is needed to fully elucidate the anti-neuroinflammatory mechanisms by *ent*-kaurane diterpenoids, these findings could be helpful in discovering potential lead compounds for the treatment of neuroinflammatory diseases.

## 3. Materials and Methods

### 3.1. General Experimental Procedures

For the isolation of the compounds from *P. multifida*, organic solvents such as methanol (MeOH), *n*-hexane, methylene chloride (CH_2_Cl_2_), chloroform (CHCl_3_), ethyl acetate (EtOAc), acetonitrile (ACN) were used (Dae Jung Pure Chemicals, Siheung, Kyunggi, Korea). Silica gel 60 was purchased from Merck (Darmstadt, Germany). Sephadex LH-20 (bead size 25–100 μm) was purchased from Pharmacia (Uppsala, Sweden). Preparative high performance liquid chromatography (HPLC) was performed using a Gilson 321 pump with a Gilson UV/Vis-151 detector (Gilson Inc., Middleton, WI, USA). JEOL LA 300 (300 MHz, JEOL Ltd., Tokyo, Japan), Bruker AMX 500 (500 MHz, Bruker, Billerica, MA, USA), and Bruker Avance 600 (600 MHz) spectrometers were used to record the nuclear magnetic resonance (NMR) spectra. Optical rotations were recorded on a JASCO P-2000 polarimeter (JASCO, Easton, MD, USA). IR spectra were obtained on a JASCO FT/IR-4200 spectrometer, and CD spectra were acquired with a Chirascan CD spectrometer (Applied Photophysics, Surrey, UK). All HR-ESI-MS data were measured on a Waters Xevo G2 QTOF mass spectrometer (Waters Co., Milford, MA, USA).

For the cell culture, Dulbecco’s modified Eagle medium (DMEM) with high glucose, fetal bovine serum (FBS) and streptomycin–penicillin (PS) were purchased from HyClone (Logan, UT, USA). 3-(4,5-dimethyl-thiazol-2yl)-2,5-diphenyltetrazolium (MTT), dimethylsulfoxide (DMSO), and lipopolysaccharide (LPS) were obtained from Sigma-Aldrich (St. Louis, MO, USA). Antibodies against cyclooxygenase-2 (COX-2) and β-actin were obtained from Santa Cruz Biotechnology (Santa Cruz, CA, USA).

### 3.2. Plant Materials

Roots of *P. multifida* were collected at the Seoul National University Forest and Baegun Mountain, Gwangyang, Korea in July 2013 and air-dried. A voucher specimen (SNU-11204) was deposited in the Herbarium of the Medicinal Plant Garden of the College of Pharmacy, Seoul National University.

### 3.3. Extraction and Isolation

The dried roots of *P. multifida* (334.0 g) were extracted three times with 80% MeOH (12 L for 90 min) in an ultrasonic apparatus. Once the solvent was removed in vacuo, the extract (30.0 g) was suspended in water and partitioned into a CH_2_Cl_2_ (7.4 g), EtOAc (2.3 g), and aqueous residue (20.1 g). The CH_2_Cl_2_ layer was suspended in 90% MeOH and then partitioned with *n*-hexane yielding a solid residue of 2.1 g (*n*-hexane) and 5.3 g (90% MeOH). The 90% MeOH fraction was subjected to silica gel column chromatography (CC) eluted with mixtures of CHCl_3_–MeOH (from 100:1 to 0:100, *v*/*v*) to yield 25 fractions (MC1~MC25). Fraction MC-8 was purified by semi-preparative HPLC using a YMC Hydrosphere C18-column with an ACN–water (50:50) solvent to yield **7** (15.1 mg). Compound **6** (1.4 mg) was isolated from MC-11 by semi-preparative HPLC (YMC Hydrosphere C18, 250 × 10 mm, ACN–water, 50:50, 4 mL/min, UV 210 nm). Compound **1** (22.2 mg) was obtained from MC-17 by recrystallization. Compound **3** (3.0 mg) was isolated from MC-20 by semi-preparative HPLC (YMC Hydrosphere C18, 250 × 10 mm, ACN–water, 50:50, 4 mL/min, UV 210 nm). The EtOAc fraction was subjected to silica gel CC eluted with mixtures of CHCl_3_–MeOH (from 40:1 to 0:100, *v*/*v*) to yield 13 fractions (EA1~EA13). Fraction EA-2 was purified by semi-preparative HPLC using a YMC Hydrosphere C18 column with an ACN–water (80:20) solvent to yield **9** (15.1 mg). Compound **12** (4.4 mg) was isolated from EA-8 by semi-preparative HPLC (YMC Hydrosphere C18, 250 × 10 mm, ACN–water, 20:80→30:70, 4 mL/min, UV 210 nm). Compound **2** (11.2 mg) was obtained from EA-11 by semi-preparative HPLC (YMC Hydrosphere C18, 250 × 10 mm, ACN–Water, 48:52, 4 mL/min, UV 210 nm). The aqueous fraction was subjected to a HP-20 column eluted with water containing increasing amounts of methanol to obtain 5 subfractions (0M, 25M, 50M, 75M, and 100M) subfraction 50M was further separated into fifteen fractions (50M-1~50M-15) using silica gel CC eluted with mixtures of CHCl_3_–MeOH (from 20:1 to 0:100, *v*/*v*). Compound **8** (38.0 mg) was obtained from the 50M-11 fraction by CC on Sephadex LH-20 with MeOH. Compounds **4** (25.4 mg), **5** (6.5 mg), and **10** (6.1 mg) were isolated from 50M-14 by semi-preparative HPLC (YMC Hydrosphere C18, 250 × 10 mm, ACN–water, 48:52, 4 mL/min, UV 210 nm). Fraction 75 M was subjected to silica gel CC eluted with mixtures of CHCl_3_–MeOH (from 30:1 to 1:1, *v*/*v*) to yield 18 fractions (75M-1~75M-18). Compound **11** (4.1 mg) was obtained from 75 M-7 by recrystallization.

### 3.4. Acid Hydrolysis of ***4***, ***10*** and ***12***

Each compound (**4**, 1.0 mg; **10**, 1.0 mg; **12**, 1.0 mg) was refluxed in 1% HCl (1 mL) for 1 h to yield aglycone and sugar. The reaction mixture was extracted with EtOAc (5 mL) to yield an aqueous fraction containing the sugar and the EtOAc fraction containing the aglycone. The aqueous fraction was concentrated and loaded onto silica gel TLC plates with EtOAc–MeOH–H_2_O–AcOH (13:3:3:4), using 20% H_2_SO_4_. The sugars of **4**, **10**, and **12** were identified as glucose by co-TLC comparison with an authentic sample. The absolute configuration of the glucose moiety in compounds **4**, **10** and **12** was determined by the method of Tanaka et al. [14] Each glucose was detected by reversed-phase HPLC using a XBridge C18-column (250 mm × 4.6 mm i.d., 5 μm, Waters) with a MeCN–H_2_O (25:75, *v*/*v*) solvent system containing 50 mM H_3_PO_4_ (flow rate 1 mL/min). The derivatives of d-glucose in **4**, **10**, and **12** were identified by comparison of their retention times with those of authentic samples treated in the same manner as described above (*t_R_*, d-glucose derivatives 12.62 min).

### 3.5. Cell Culture

The BV-2 mouse microglial cells were generously provided by Dr. Sun Yeou Kim at Kyunghee University (Suwon, Korea). The cells were maintained in DMEM containing 10% FBS with 1% PS at 37 °C in a humidified atmosphere of 95% air and 5% CO_2_. All tested fractions and samples were dissolved in DMSO (final concentration in cultures ≤0.1%).

### 3.6. NO Production Assay and Cell Viability Assay

To evaluate NO production, we seeded BV-2 cells in 96-well plates (4 × 10^5^ cells/mL) for 24 h and then treated them with various concentrations of the samples for 1 h before exposure to 100 ng/mL of LPS. After the 24 h incubation, the nitrite in the culture media was measured to assess NO production in the BV-2 cells with the Griess reagent (1% sulfanilamide and 0.1% naphtylethylenediamine dihydrochloride in 2% phosphoric acid). In 96-well plates, 100 μL of sample aliquots were mixed with 100 μL of Griess reagent and incubated at room temperature for 15 min. The absorbance at 550 nm was measured on a microplate reader. The concentration was determined with a nitrite standard curve. Cell viability was assessed by the MTT assay. After exposing the cells to 2 mg/mL MTT for 2 h, the absorbance was measured by an ELISA plate reader at 562 nm.

### 3.7. PGE_2_, TNF-α, IL-1β, and IL-6 ELISA Assay

BV-2 microglial cells were cultured in 96-well plates, preincubated for 30 min. with different concentrations of compounds **1** and **7** and then stimulated for 24 h with LPS (Sigma-Aldrich Co.). Supernatant from the culture (100 μL) was collected to determine the concentration of PGE_2_, TNF-α, IL-1β, and IL-6, respectively, with ELISA kits (TNF-α, Abcam, Cambridge, MA, USA; PGE_2_, IL-1β, and IL-6, R&D Systems, Minneapolis, MN, USA).

### 3.8. Western Blot Analysis

Cells were harvested and lysed with radioimmunoprecipitation (RIPA) buffer (Bio-Rad, Hercules, CA USA). Cell lysates were centrifuged, and the supernatant was collected. The protein concentration was determined by protein assay reagents (Bio-Rad). The proteins were separated onto 10% SDS-polyacrylamide gel and transferred to polyvinylidene fluoride (PVDF) membrane. The membranes were blocked with 5% non-fat dry milk in Tris-buffered saline with 0.1% Tween-20 (TBS-T) buffer for 1 h at room temperature and then incubated with primary antibodies in TBS-T buffer containing 1% non-fat dry milk overnight at 4 °C. The membranes were washed with TBS-T buffer and incubated with goat anti-rabbit or anti-mouse horseradish peroxidase-conjugated IgG secondary antibodies from Santa Cruz Biotechnology (Santa Cruz, CA, USA) for 2 h. The membranes were washed with TBS-T buffer, and the antigen-antibody complex was detected with an enhanced chemiluminescence detection system (GE Healthcare, Milwaukee, MI, USA).

### 3.9. Statistical Analysis

Data were evaluated for statistical significance with one way ANOVA using the IBM SPSS Statistics 23 package. The data were considered statistically significant with * *p* < 0.05, ** *p* < 0.01, and *** *p* < 0.001.

## Figures and Tables

**Table 1:** ^1^H- and ^13^C-NMR spectral data of **4**, **10** and **12** in CD_3_OD (δ in ppm; *J* in Hz).

Position	4 ^a^		10 ^b^		12 ^a^	
	δ_H_ (*J* in Hz)	δ_C_	δ_H_ (*J* in Hz)	δ_C_	δ_H_ (*J* in Hz)	δ_C_
1a, 1b	2.30, m, 0.76, t (11.9)	49.1	2.28, m, 0.77, t (11.9)	49.4	2.28, m, 0.77, t (11.4)	50.1
2	4.08, m	74.9	4.02, m	75.0	4.01, m	74.9
3a, 3b	1.72, m, 1.37, m	43.0	1.88, m, 1.13, m	49.1	1.87, m, 1.12, m	49.6
4		40.7		36.4		36.4
5	1.20, m	50.5	0.82, m	58.0	0.85, m	58.0
6a, 6b	1.55, m, 1.31, m	20.7	1.66, m, 1.38, m	22.0	1.59, m, 1.35, m	22.0
7a, 7b	1.63, m, 1.52, m	36.8	1.65, m, 1.46, m	43.5	1.60, m, 1.50, m	43.9
8		49.7		47.2		46.5
9	1.10, m	56.6	1.04, m	59.1	1.06, m	59.0
10		42.9		42.9		42.9
11a, 11b	1.65, m, 1.48, m	20.1	1.66, m, 1.58, m	20.2	1.68, m, 1.56, m	20.3
12a, 12b	1.68, m, 1.46, m	34.7	1.58, m	28.6	1.65, m, 1.55, m	28.1
13	2.70, br s	44.5	2.11, br s	48.2	2.02, br s	47.2
14a, 14b	1.90, d (11.6), 1.38, m	38.2	1.82, d (11.5), 1.73, dd (11.5, 4.1)	39.0	1.90, m, 1.61, m	39.1
15, 15b	3.76, br s	84.6	1.88, d (14.2), 1.41, d (14.2)	57.4	1.53, m, 1.40, m	54.8
16		161.3		89.3		83.6
17, 17b	5.17, s, 5.06, s	109.8	1.38, s	22.2	3.69, d (11.4), 3.58, d (11.4)	67.7
18, 18b	3.37, d (11.0), 3.06, d (11.0)	72.7	0.92, s	35.1	0.93, s	35.0
19	0.79, s	19.5	0.86, s	23.5	0.86, s	23.5
20	1.13, s	20.6	1.09, s	20.2	1.09, s	20.2
1*’*	4.38, d (7.8)	103.3	4.36, d (7.8)	103.4		103.3
2*’*	3.13, t (8.3)	75.9	3.10, t (8.2)	76.0		76.0
3*’*	3.26, m	78.6	3.26, m	78.6		78.6
4*’*	3.27, m	72.5	3.27, m	72.5		72.5
5*’*	3.35, m	78.9	3.34, m	79.1		78.9
6*’*a, 6*’*b	3.85, m, 3.66, dd (11.0, 3.8)	63.6	3.85, m, 3.78, m	63.6		63.6
1″			4.36, d (7.8)	100.1		
2″			3.12, t (8.3)	75.9		
3″			3.20, m	78.4		
4″			3.26, m	72.6		
5″			3.35, m	78.9		
6″a, 6″b			3.66, m, 3.62, m	63.5		

^a^ ^1^H-NMR data were measured at 500 MHz, and ^13^C-NMR data were measured at 125 MHz in CD_3_OD, respectively; ^b^ ^1^H-NMR data were measured at 600 MHz, and ^13^C-NMR data were measured at 150 MHz in CD_3_OD, respectively.

**Table 2:** Inhibitory activity of compounds **1**–**12** on NO production in LPS-activated BV-2 cells.

Compound	IC_50_^a^ (μM)	Cell Viability ^b^ (%)	Compound	IC_50_^a^ (μM)	Cell Viability ^b^ (%)
**1**	13.9	90.7± 7.3	**7**	10.8	88.7 ± 2.4
**2**	92.0	95.7 ± 4.0	**8**	101.4	91.7 ± 3.7
**3**	84.0	87.7 ± 2.9	**9**	121.7	97.5 ± 1.3
**4**	148.1	90.5 ± 10.2	**10**	182.2	88.2 ± 8.0
**5**	147.9	73.6 ± 2.9	**11**	171.3	83.2 ± 10.2
**6**	137.1	85.9 ± 0.1	**12**	113.7	93.6 ± 8.3
			l-NAME ^c^	53.5	100.3 ± 5.0

^a^ IC_50_ value of each compound was defined as the concentration (μM) that caused 50% inhibition of NO production in LPS-stimulated BV-2 cells; ^b^ Cell viability was measured by the MTT assay after treatment with 100 μM of each compound for 24 h. Results are expressed as the mean ± SD. ^c^ N_ω_-nitro-l-arginine methyl ester (L-NAME) was used as a positive control.

## Supplementary Materials

Supplementary materials can be accessed at: http://www.mdpi.com/1420-3049/22/1/27/s1.
