# Supplementary material for: Anti-Neuroinflammatory ent-Kaurane Diterpenoids from Pteris multifida Roots
Source: Molecules. 2016 Dec 26;22(1):27. doi: 10.3390/molecules22010027 (PMC6155576; doi:10.3390/molecules22010027)
Supplement: Supplementary file 1 [file molecules-22-00027-s001.pdf]

Supplementary Materials: Anti-Neuroinflammatory *ent*-Kaurane Diterpenoids from *Pteris multifida* Roots

Jung Wha Kim, Ji Yeon Seo, Won Keun Oh and Sang Hyun Sung

Table S1. ^{1}H-NMR data of compounds **1–3**, **5–9** and **11** (δ in ppm, J values in parentheses).

Position	1 [a]	2 [b]	3 [b]	5 [c]	6 [b]	7 [b]	8 [b]	9 [b]	11 [b]
					δ_H (*J* in Hz)				
1a, 1b	2.16, m, 0.65, t (11.9)	2.30, m, 0.77, t (11.8)	2.16, m, 0.65, t (11.6)	2.31, m, 0.81, t (11.9)	2.11, m, 0.67, t (11.7)	2.13, m, 0.66, t (11.6)	2.26, m, 0.79, t (11.8)	2.14, m, 0.64, t (11.6)	2.14, d (9.75), 0.64, t (11.6)
2	3.85, m	4.01, m	3.91, m	3.99, m	3.83, m	3.83, m	3.99, m	3.85, m	3.92, m
3a, 3b	1.73, m, 1.08, m	1.89, m, 1.14, m	1.58, m, 1.37, m	2.28, m, 0.89, t (11.8)	1.64, m, 1.12, m	1.72, m, 1.08, m	1.88, m, 1.15, m	1.71, m, 1.05, m	1.55, m, 1.36, m
5	0.81, m	0.85, m	1.17, m	0.98, d (11.9)	0.94, d (10.1)	0.87, m	0.91, m	0.80, m	1.17, m
6	1.62, m	1.62, m	1.56, m, 1.32, m	1.56, m, 1.32, m	3.91, td (10.1, 3.7)	1.66, m, 1.41, m	1.66, m, 1.38, m	1.58, m, 1.35, m	1.48, m, 1.33, m
7, 7b	1.66, m, 1.46, m	1.66, m, 1.47, m	1.62, m, 1.51, m	1.63, m, 1.52, m	1.86, m, 1.62, m	2.33, m, 1.21, m	2.34, m, 1.20, m	1.60, m, 1.47, m	1.55, m
9	1.06, m	1.06, m	1.10, m	1.08, m	1.02, m	1.27, d (8.5)	1.27, m	1.03, m	1.07, m
11, 11b	1.60, m, 1.20, m	1.67,m 1.47, m	1.65, m, 1.50, m	1.63, m, 1.52, m	1.62, m	1.57, m, 1.38, m	1.59, m, 1.40, m	1.60, m, 1.40, m	1.61, m
12, 12b	1.68, m, 1.49, m	1.67,m 1.47, m	1.68, m, 1.48, m	1.69, m, 1.48, m	1.71, m, 1.48, m	1.81, m, 1.60, m	1.80, m, 1.59, m	1.80, m, 1.59, m	1.59, m
13	2.70, br s	2.70, br s	2.70, br s	2.70, br s	2.69, br s	2.75, br s	2.75, br s	1.82, br s	1.82, br s
14, 14b	1.90, m, 1.38, m	1.90, m, 1.37, m	1.91, d (11.8), 1.38, d (12.0)	1.87, d (11.5), 1.37, m	1.82, m, 1.50, m	4.13, s	4.13, s	1.89, m, 1.60, m	1.91, d (11.6), 1.60, m
15	3.75, s	3.75, s	3.76, br s	3.74, br s	3.80, br s	3.74, s	3.74, s	1.53, m	1.54, s
17, 17b	5.17 s, 5.07 s	5.17, s, 5.06, s	5.17, s, 5.06, s	5.17, s, 5.06, s	5.19, s, 5.10, s	5.32, s, 5.17, s	5.32, s, 5.16, s	1.33, s	1.33, s
18, 18b	0.94, s	0.95, s	3.35, d (11.3) 3.05, d (11.0)	3.64, m, 3.31, m	1.20, s	0.96, s	0.97, s	0.92, s	3.35, d (10.9), 3.02, d (11.0)
19	0.88, s	0.88, s	0.78, s	1.01, s	1.08, s	0.89, s	0.89, s	0.89, s	0.76, s
20	1.10, s	1.10, s	1.13, s	1.09, s	1.10, s	1.04, s	1.04, s	1.09, s	1.12, s
Glucose									
1'				4.42, d (7.8)			4.36, d (7.8)		
2'				3.11, m			3.12, t		
3'				3.27, m			3.26, m		
4'				3.26, m			3.27, m		
5'				3.34, m			3.35, m		
6'				3.85, m, 3.66, m			3.84, m, 3.65, dd		

^{1}H-NMR data were measured at [a] 300, [b] 500, and [c] 600 MHz in CD_3OD, respectively.

Table S2. ^{13}C-NMR data of compounds **1–3**, **5–9** and **11** (*δ* in ppm, *J* values in parentheses).

Position	1 [a]	2 [b]	3 [b]	5 [c]	6 [b]	7 [b]	8 [b]	9 [b]	11 [b]
1	51.2	49.5	50.9	49.6	51.5	51.1	49.4	51.2	50.8
2	66.3	74.9	66.3	74.1	66.0	66.3	74.9	66.3	66.3
3	52.6	49.3	46.2	42.8	54.6	52.5	49.2	52.7	46.3
4	36.4	36.3	40.8	42.2	37.3	36.4	36.3	36.5	40.9
5	57.8	58.0	50.4	58.6	62.3	57.8	57.9	57.9	50.3
6	20.2	20.0	20.7	21.1	70.1	20.7	20.6	22.0	21.6
7	37.3	37.3	36.8	37.8	46.4	29.7	29.7	43.9	43.4
8	49.7	49.9	49.7	50.0	50.0	54.4	54.4	47.1	47.1
9	56.7	56.7	56.6	56.9	56.1	58.8	58.8	59.1	59.0
10	43.2	43.2	42.9	43.0	44.6	43.1	43.1	42.9	42.7
11	21.1	21.1	20.1	20.1	19.7	19.6	19.6	20.0	20.5
12	34.7	34.7	34.7	34.6	34.6	34.5	34.5	28.7	28.7
13	44.5	44.5	44.5	44.5	43.8	52.4	52.5	50.4	50.4
14	38.2	38.2	38.3	38.1	38.3	78.1	78.1	39.4	39.4
15	84.6	84.6	84.7	84.6	84.6	84.8	84.8	59.5	59.5
16	161.2	161.3	161.3	161.2	161.2	159.9	159.9	80.6	80.6
17	109.8	109.8	109.8	109.8	110.4	112.5	112.5	25.2	25.3
18	35.1	35.1	72.7	29.0	38.1	35.1	35.2	35.0	72.6
19	23.7	23.7	19.6	66.4	24.1	23.7	23.7	23.6	19.5
20	20.1	20.1	20.6	20.7	20.9	20.3	20.2	20.2	20.7
glucose									
1′		103.4		103.2			103.4		
2′		75.9		76.0			75.9		
3′		78.6		78.6			78.6		
4′		72.5		72.6			72.5		
5′		78.9		78.9			78.8		
6′		63.6		63.6			63.6		

^{13}C-NMR data were measured at [a] 75, [b] 125, and [c] 150 MHz in CD_3OD, respectively.

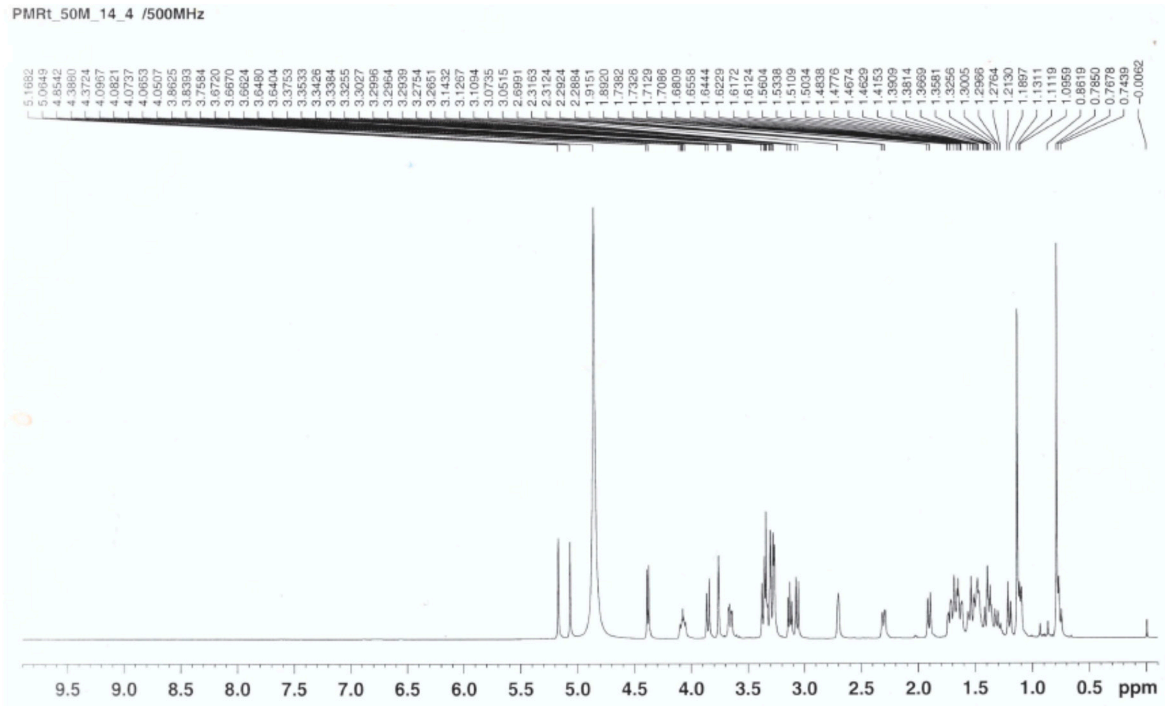

Figure S1. The ^{1}H-NMR spectrum of **4**.

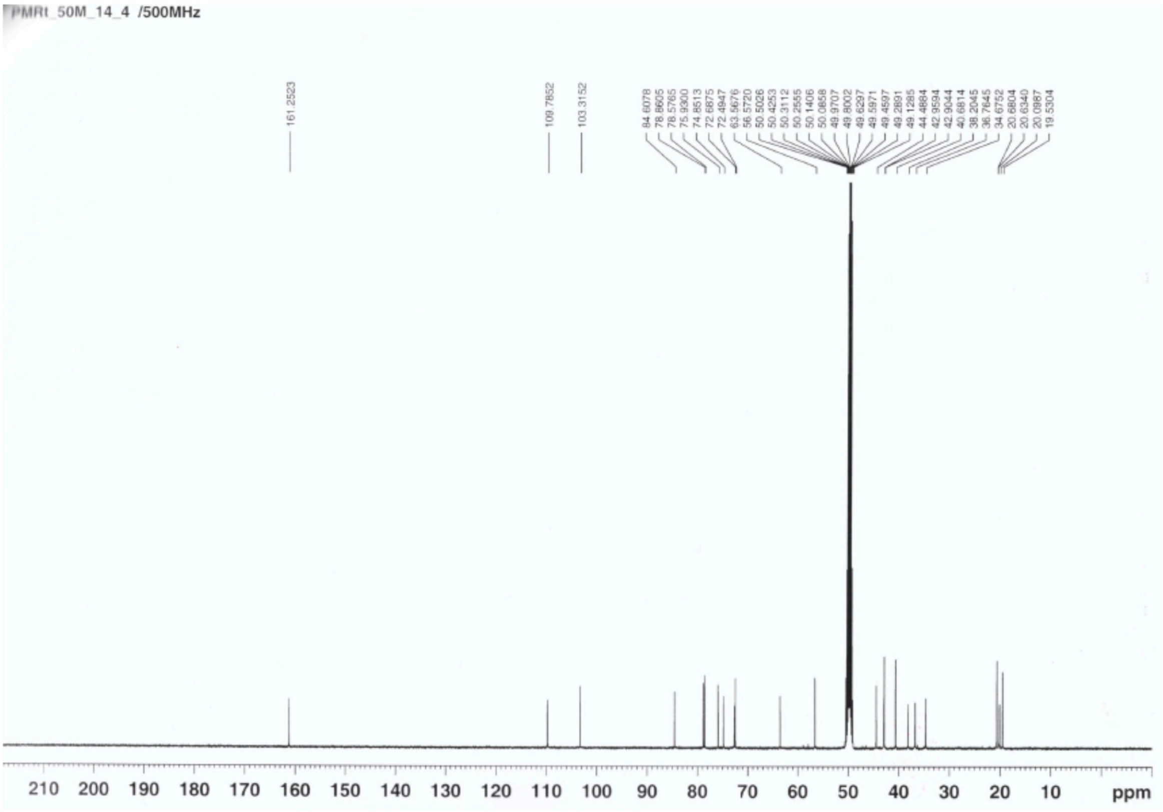

Figure S2. The ^{13}C-NMR spectrum of **4**.

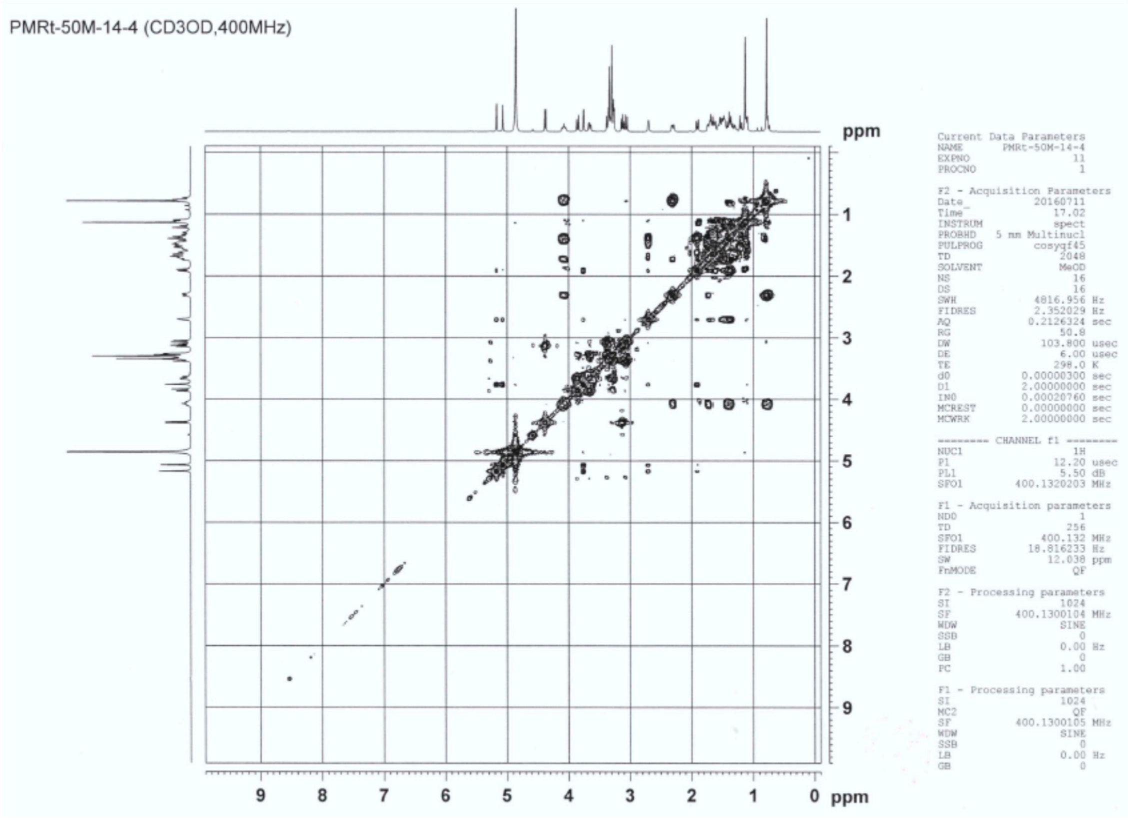

Figure S3. The ^{1}H-^{1}H COSY spectrum of **4**.

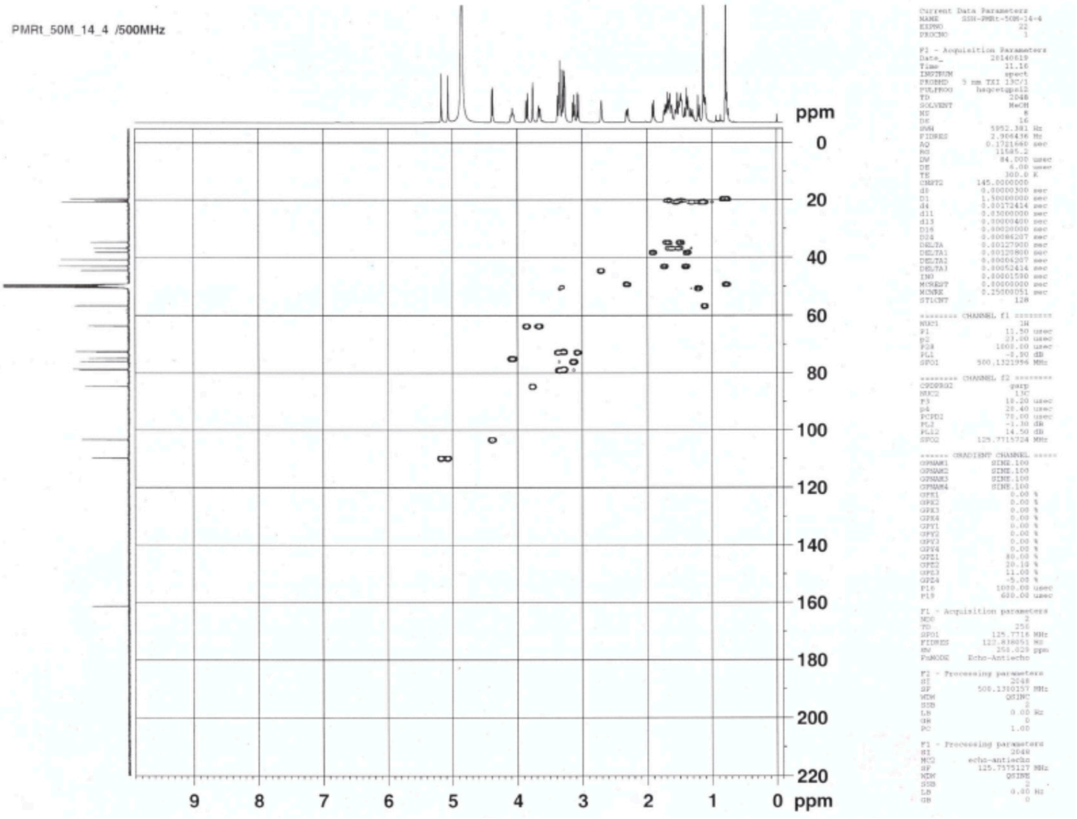

Figure S4. The HMQC spectrum of **4**.

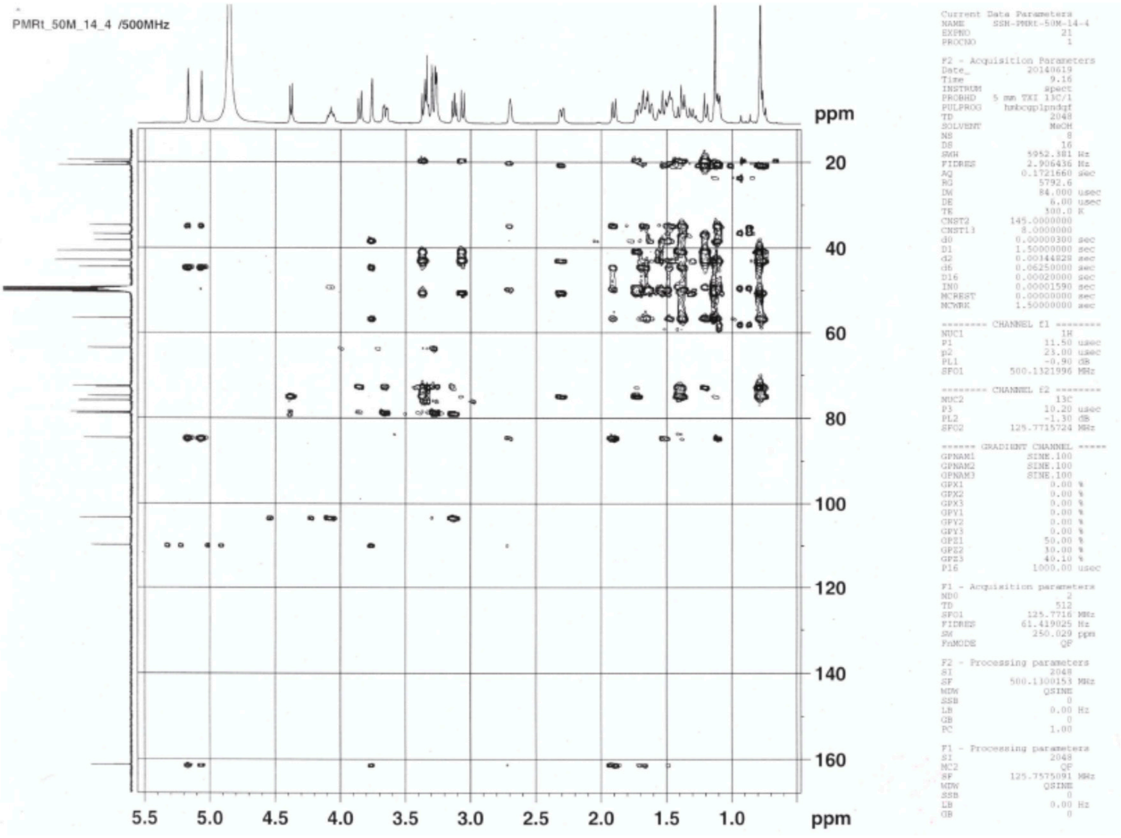

Figure S5. The HMBC spectrum of **4**.

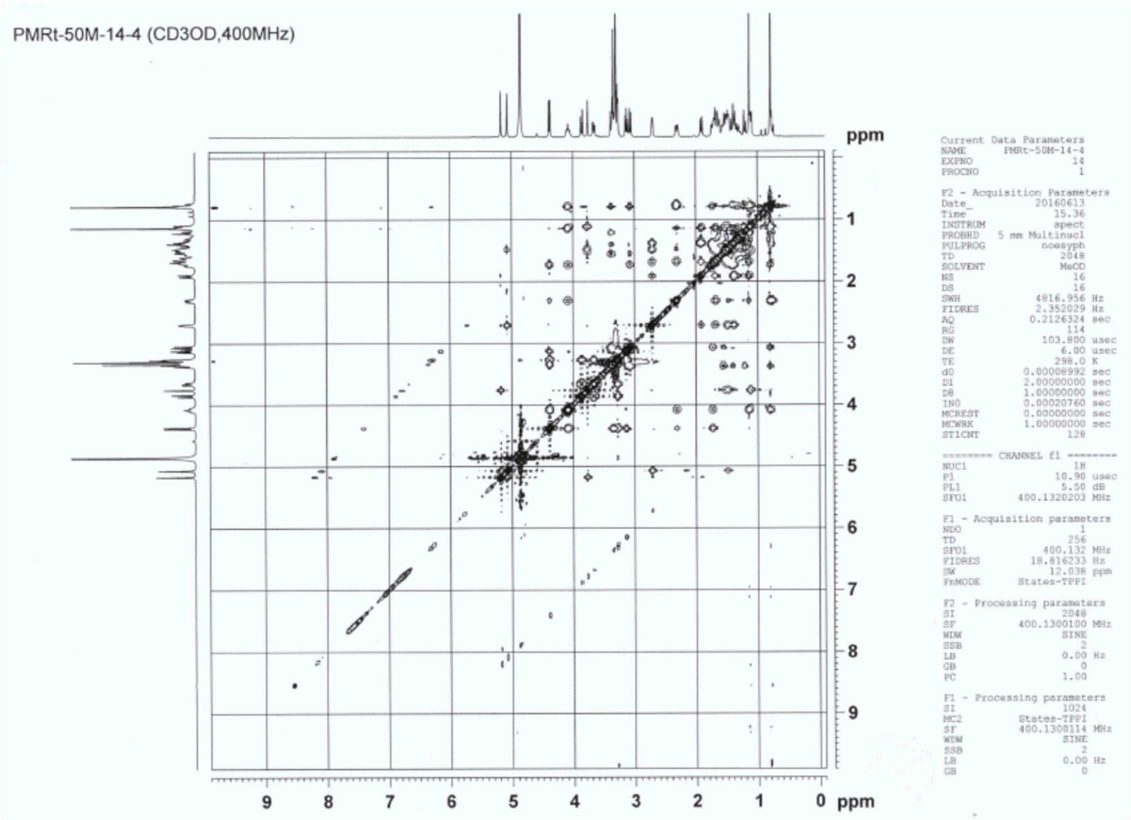

Figure S6. The NOESY spectrum of **4**.

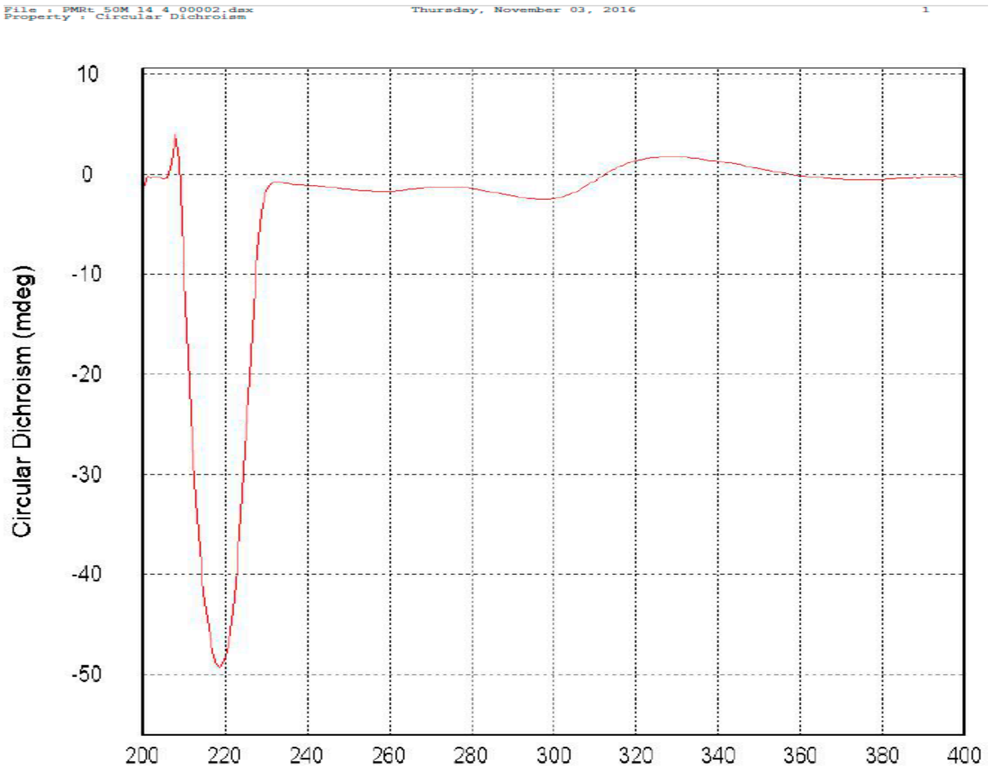

Figure S7. The CD spectrum of **4**.

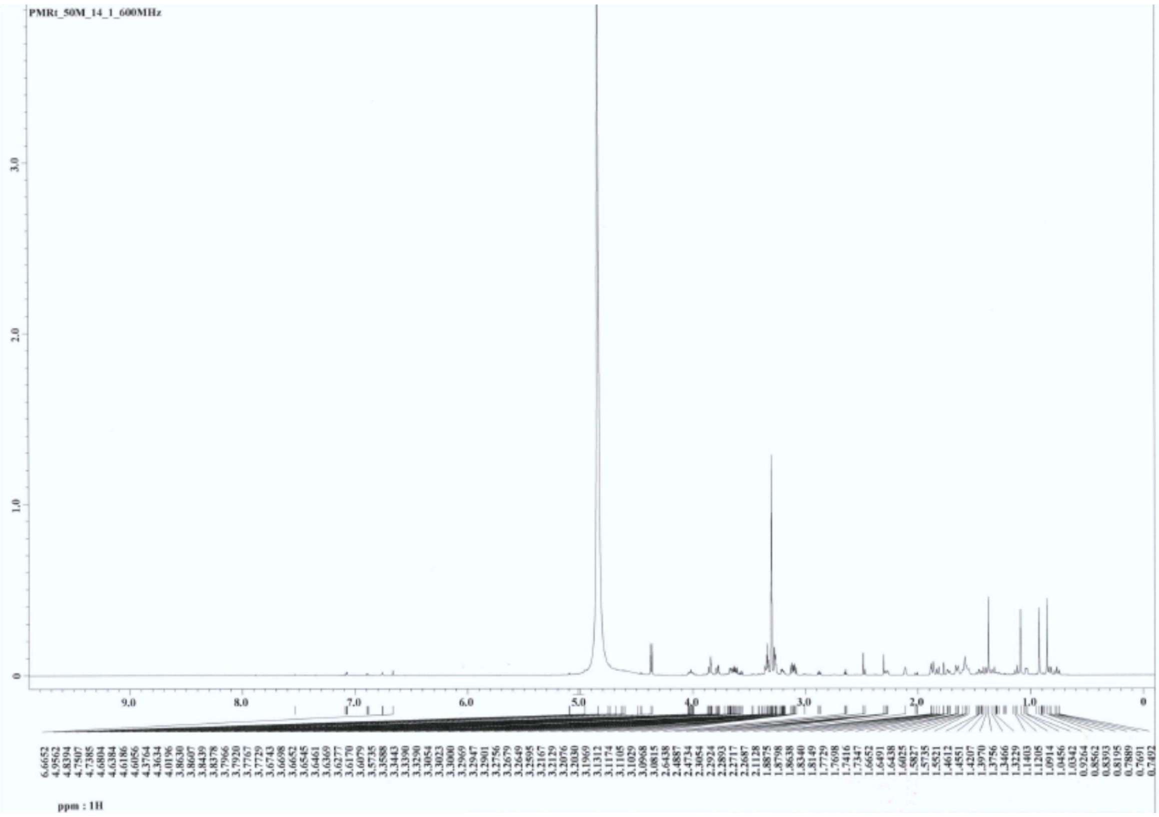

Figure S8. The ^{1}H-NMR spectrum of **10**.

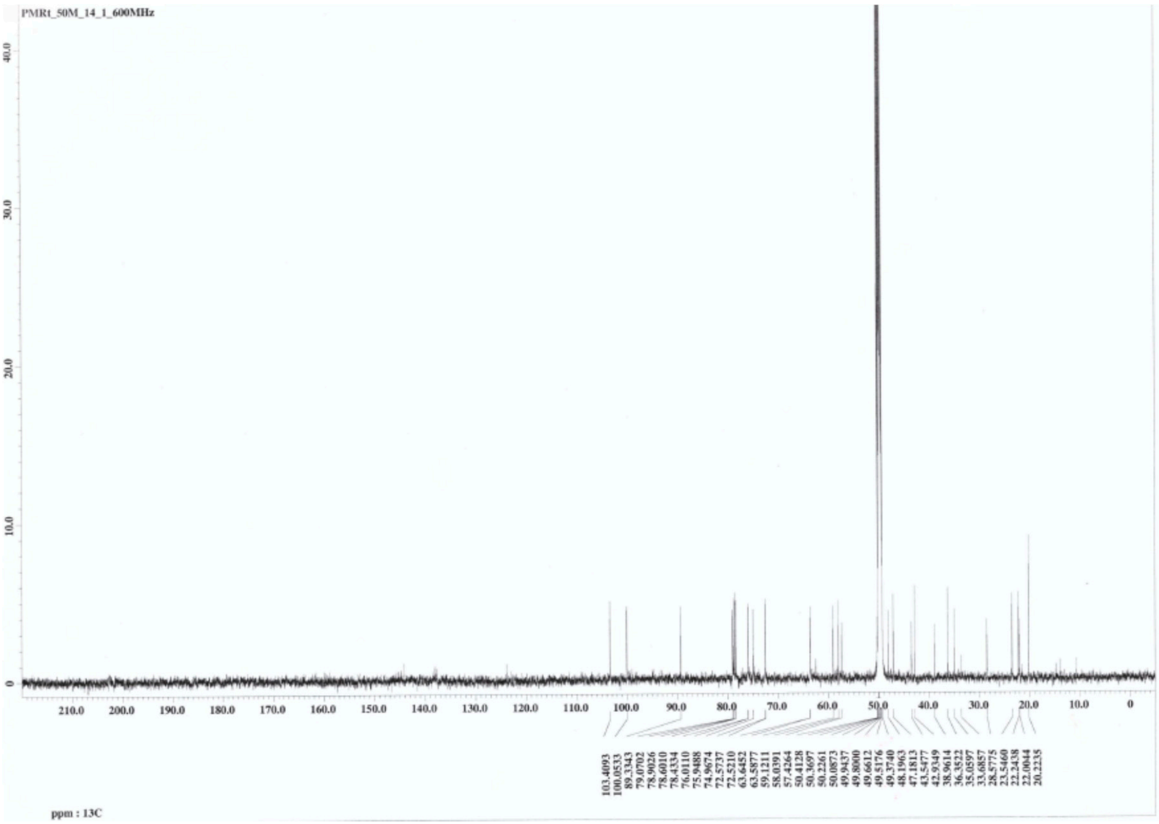

Figure S9. The ^{13}C-NMR spectrum of **10**.

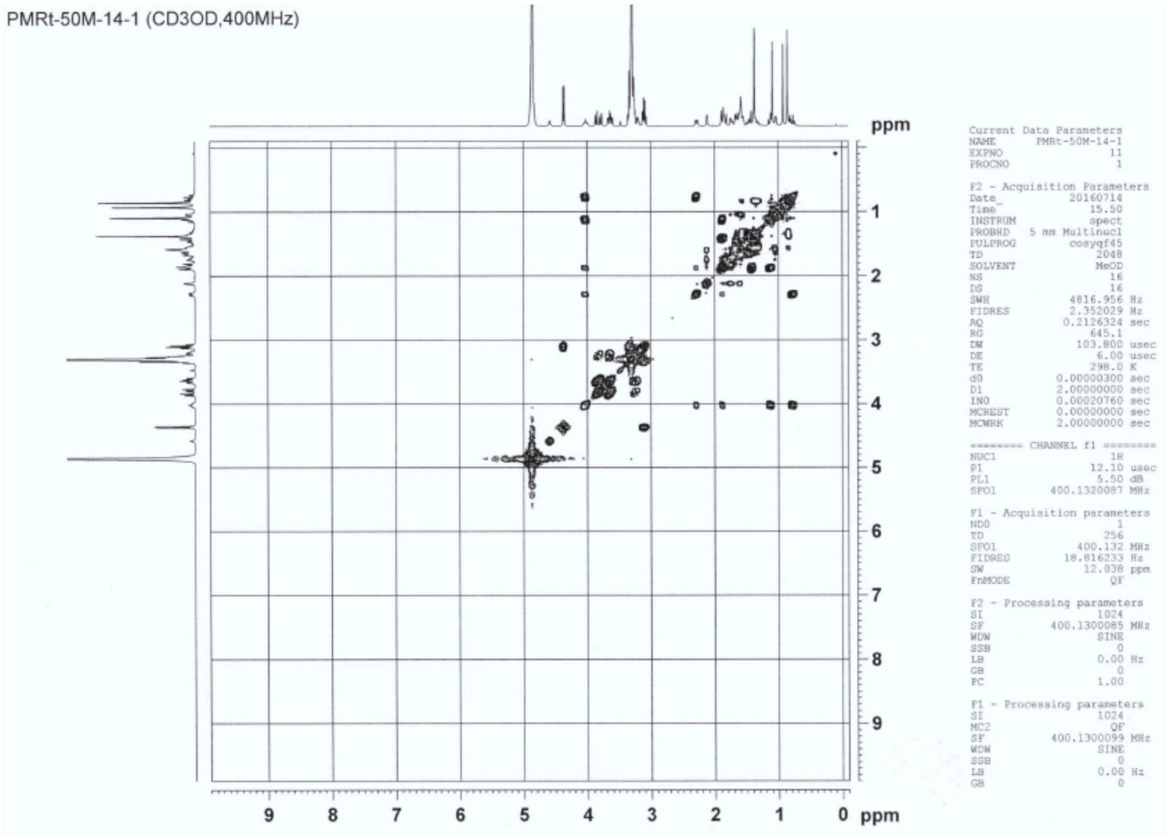

Figure S10. The ^{1}H-^{1}H COSY spectrum of **10**.

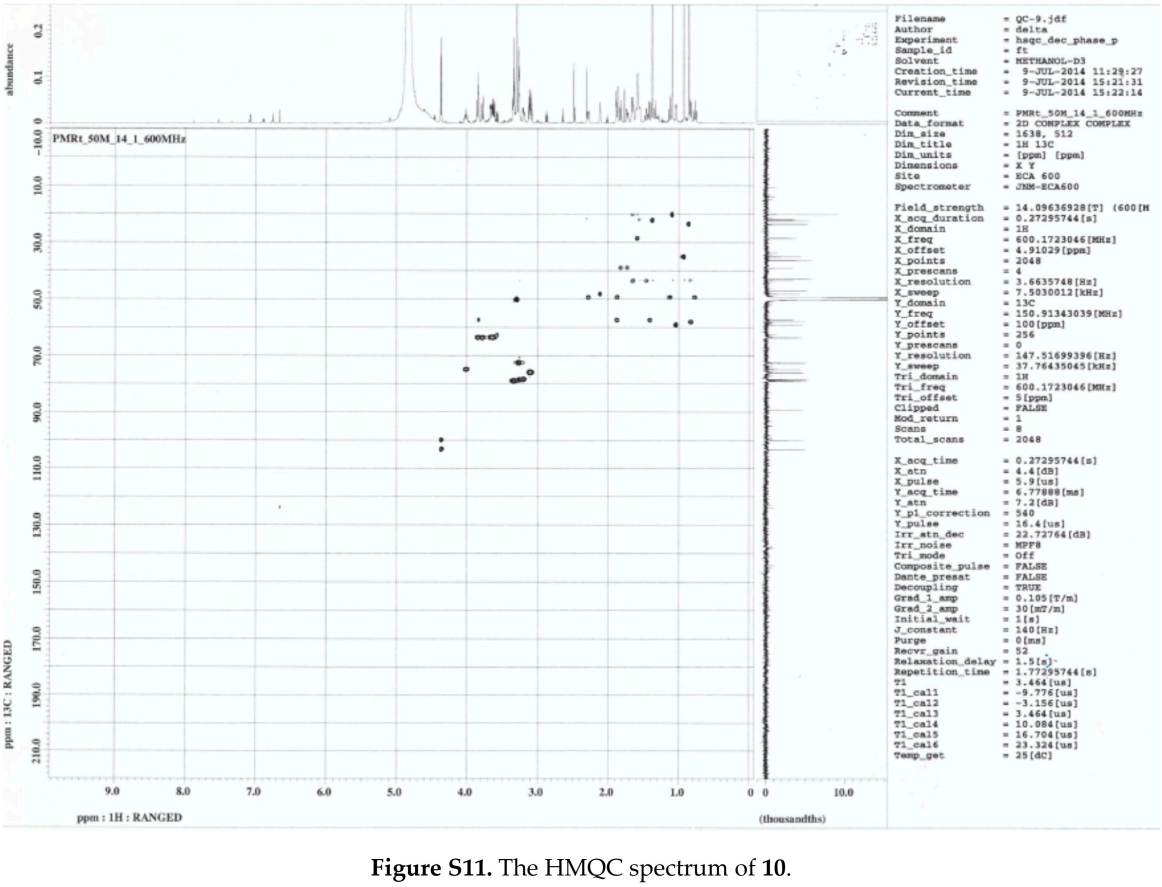

Figure S11. The HMQC spectrum of **10**.

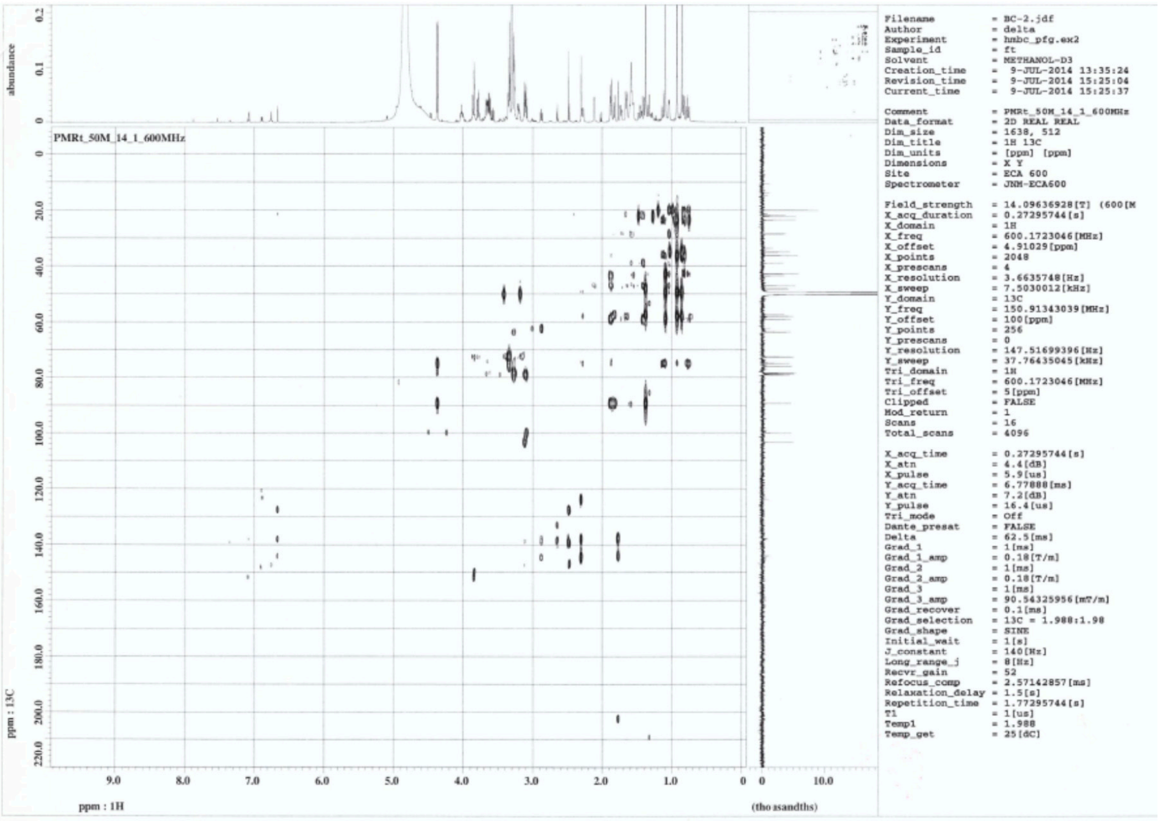

Figure S12. The HMBC spectrum of **10**.

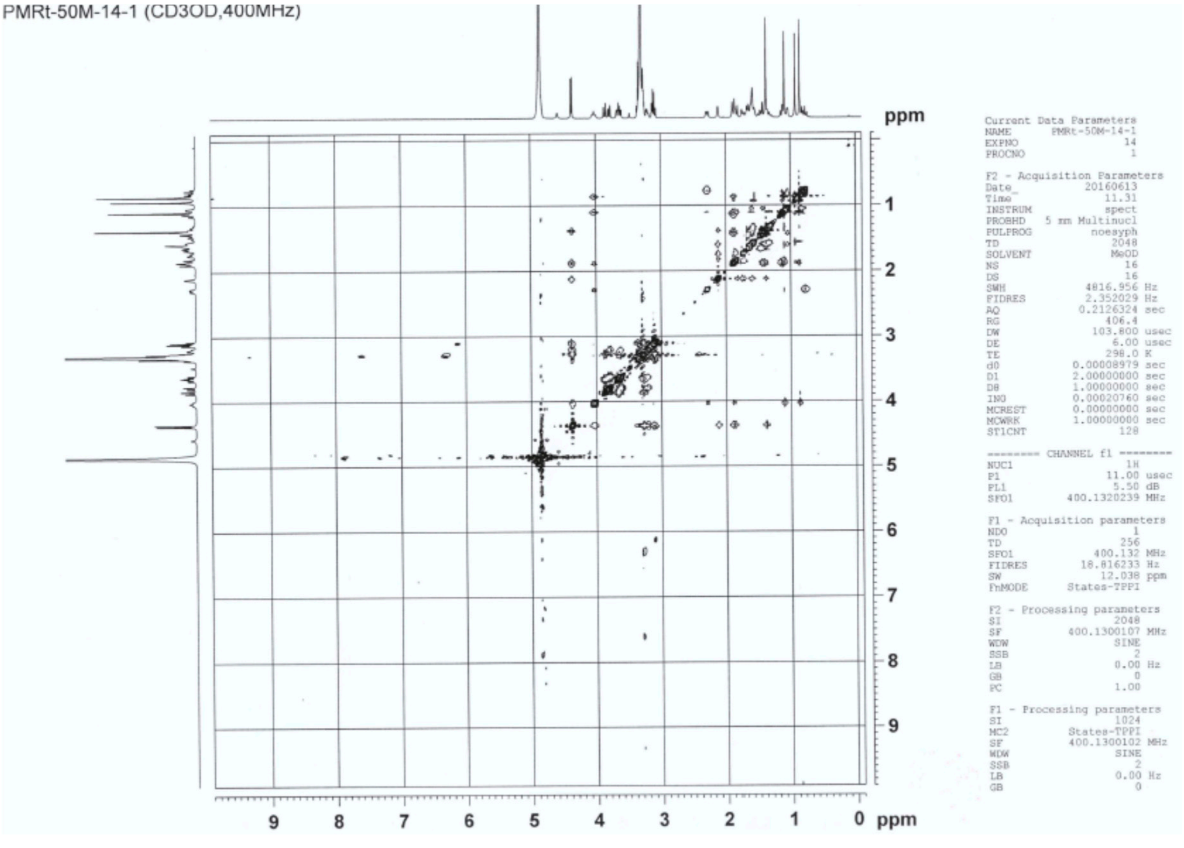

Figure S13. The NOESY spectrum of **10**.

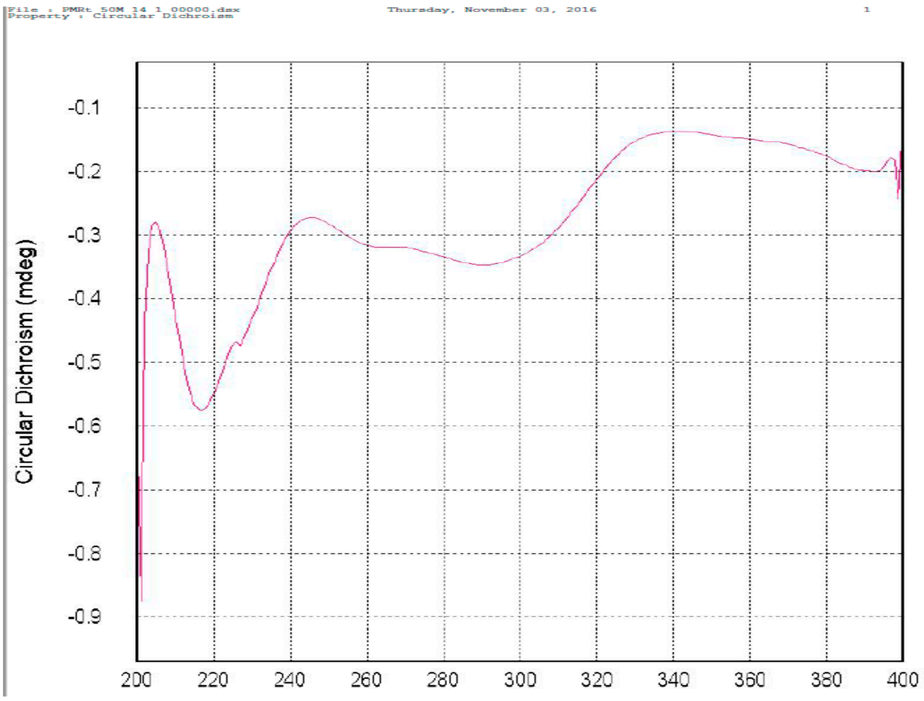

Figure S14. The CD spectrum of compound **10**.

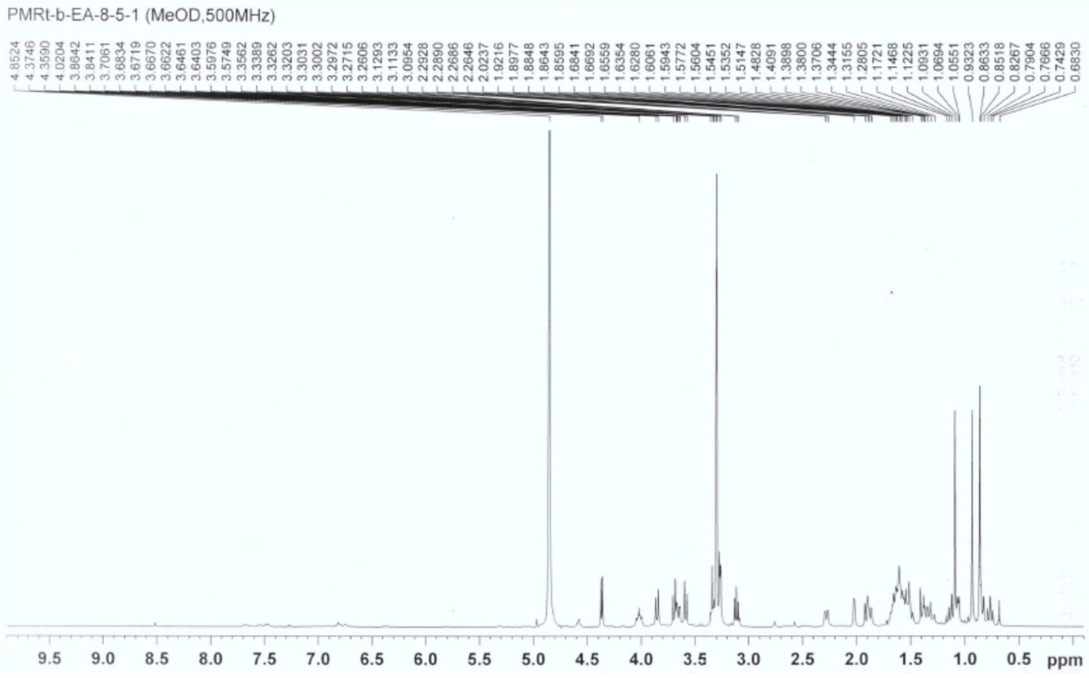

Figure S15. The ^{1}H-NMR spectrum of **12**.

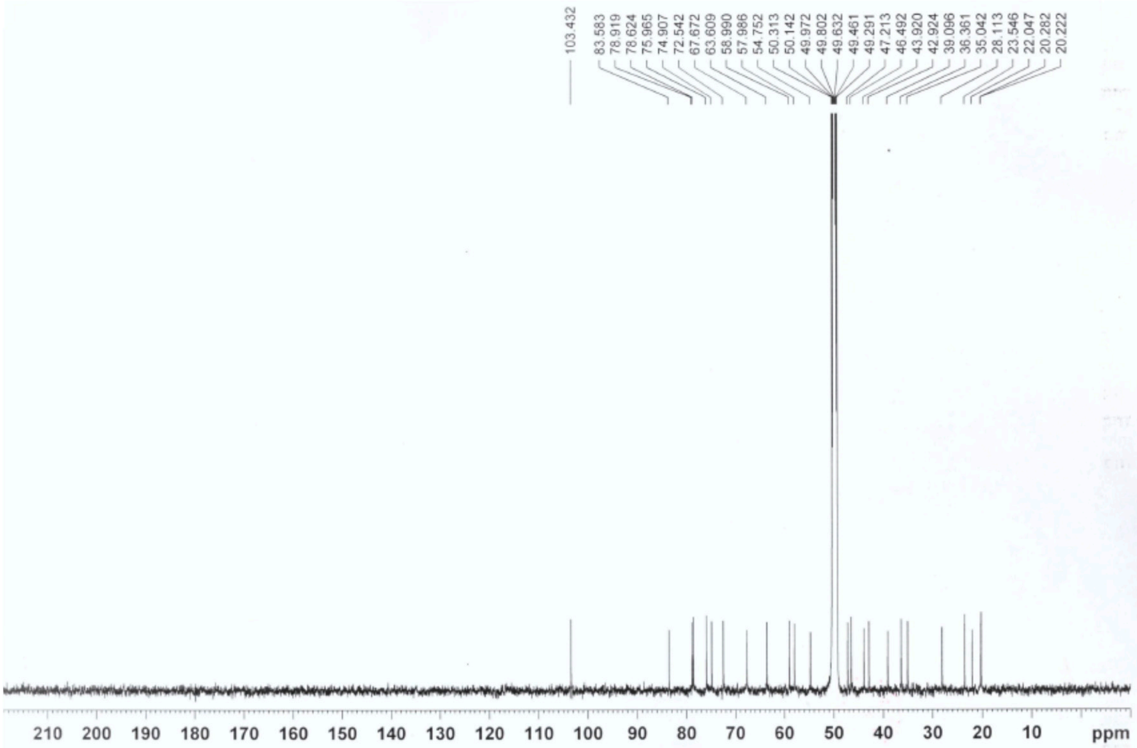

Figure S16. The ^{13}C-NMR spectrum of **12**.

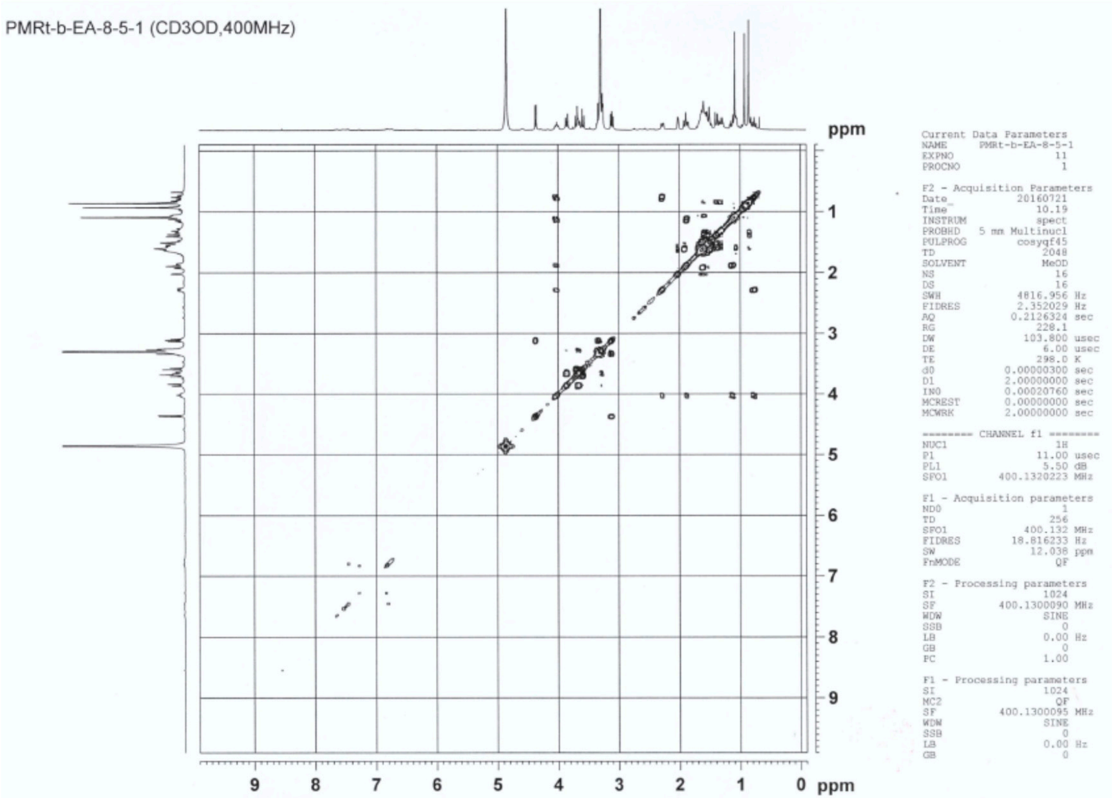

Figure S17. The ^{1}H-^{1}H COSY spectrum of **12**.

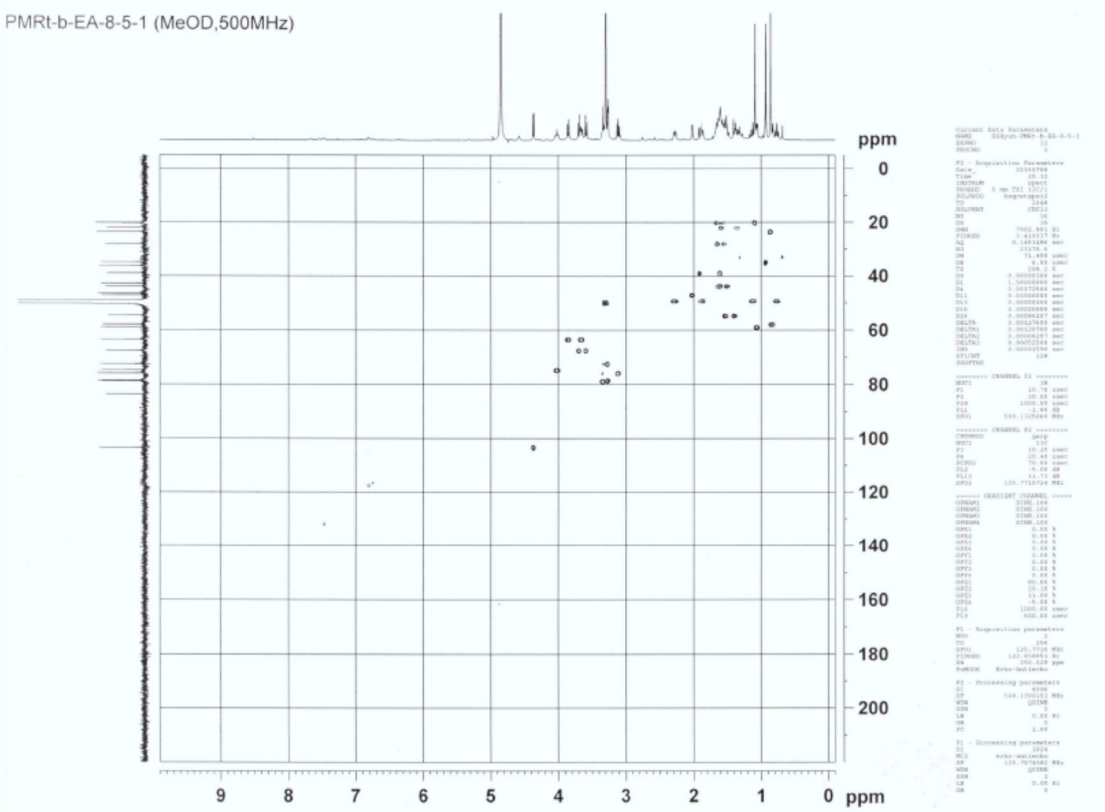

Figure S18. The HMQC spectrum of **12**.

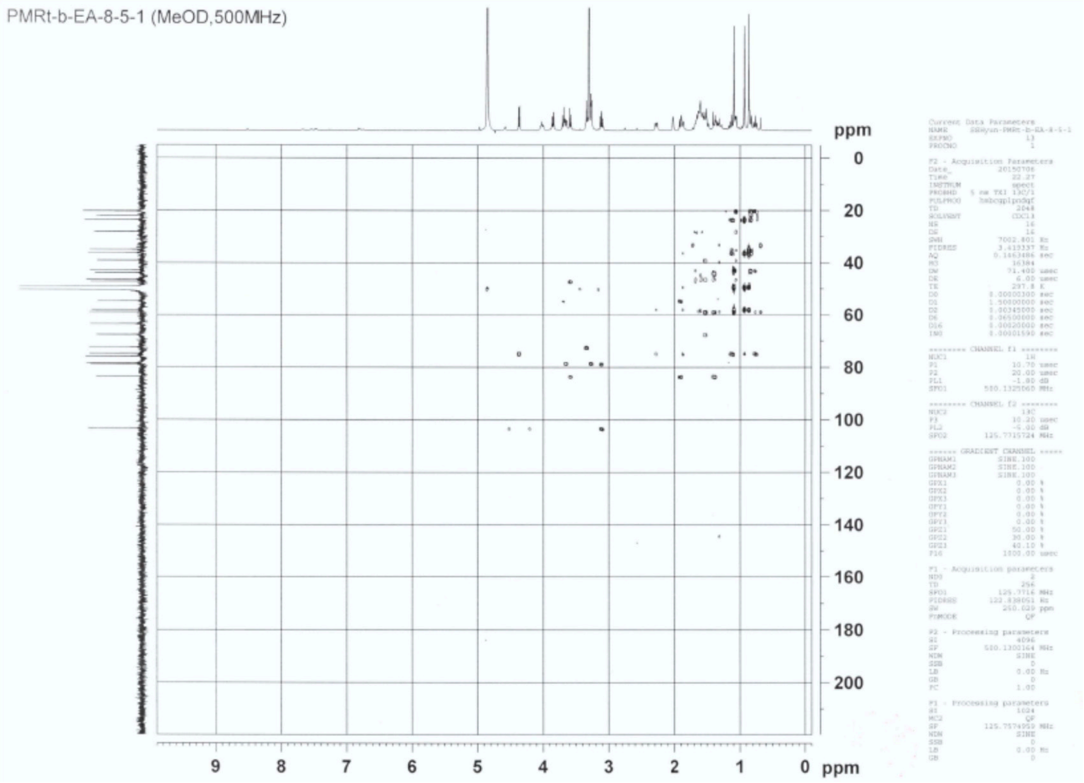

Figure S19. The HMBC spectrum of **12**.

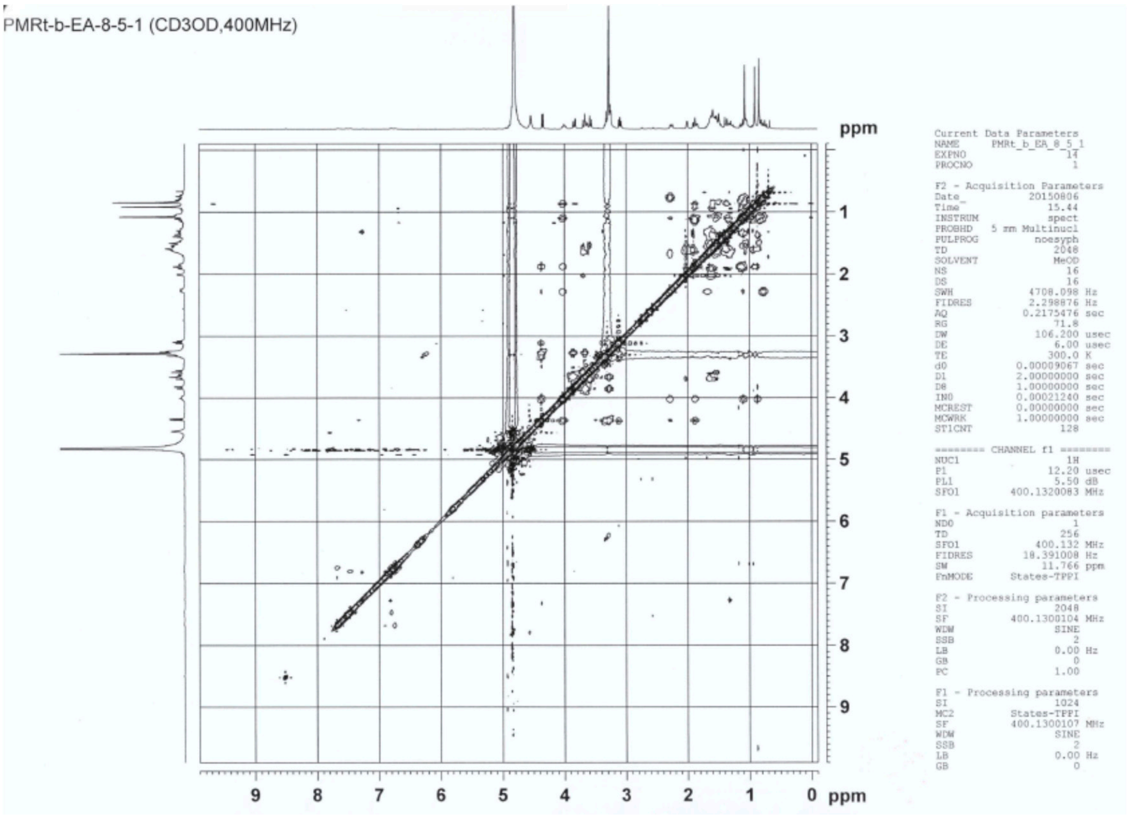

Figure S20. The NOESY spectrum of **12**.

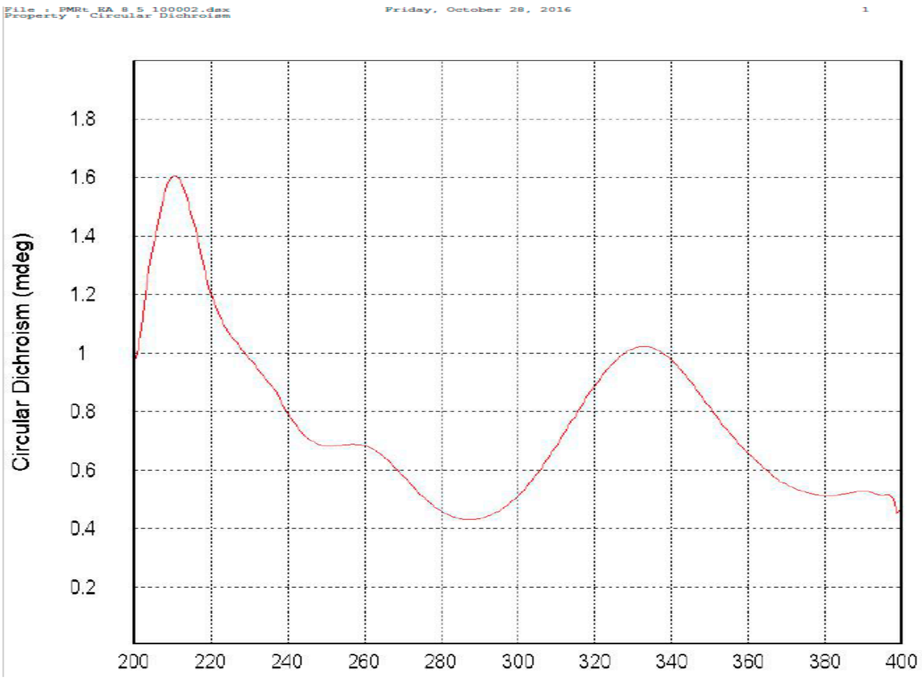

Figure S21. The CD spectrum of **12**.
